# Addressing Power in Local-Level Policies and Programs to Reduce Health Inequities – A Systematic Review

**DOI:** 10.1177/27551938251401131

**Published:** 2025-12-01

**Authors:** Sally Schultz, Anne Barrow, Sharon Friel, Christina Zorbas, Anna Peeters, Kathryn Backholer

**Affiliations:** 1 612996 Deakin University, Institute for Health Transformation, Global Centre for Preventive Health and Nutrition, School of Health and Social Development, Faculty of Health, Geelong, Australia; 2 Menzies Centre for Health Governance, School of Regulation and Global Governance, 2219 Australian National University, Canberra, Australia

**Keywords:** health equity, power, health interventions, policy

## Abstract

Health inequities are driven by the unequal distribution of resources and power. Local-level actors are closely connected to communities and have the potential to address unfair imbalances in power through health equity interventions. Yet practical strategies on how to do this remain unclear. To address this gap, we conducted a systematic review of five databases, examining how power was addressed in the design and implementation of local-level health equity interventions and their reported impacts. Thirty-eight international studies were analysed using the Health Equity Power Framework and Four Expressions of Power typology. Most articles described community organizing, health education, advocacy, and community funding initiatives. Interventions that strengthened community knowledge, connectedness, and leadership rebalanced power by enhancing individual and collective agency. Shifts in rigid, inequitable structures and institutional processes were observed when interventions activated multiple types of power, across different forms and spaces. Interventions informed by power-centered frameworks and principles, such as empowerment theory and self-determination, helped actors rebalance power dynamics, while entrenched structural and institutional power imbalances moderated efforts to rebalance power. This review underscores the role of local governments, institutions, and community actors in addressing power imbalances and provides practical guidance on strategies to support equitable policymaking.

The existence of health inequities is a critical global human rights issue.^1,2^ Health inequities are avoidable and unjust differences in health outcomes arising from systemic inequalities in factors such as socioeconomic status, occupation, education, ethnicity, place of residence, gender, disability, or sexual orientation.^3^ Health inequities are observed globally for a range of health outcomes including life expectancy, mortality, and prevalence of non-communicable diseases.^4–9^ Structural determinants of health inequities, such as public, social, and macroeconomic policies, and dominant societal values shape the unequal distribution of power and resources that perpetuate health inequities.^10,11^ Government, institutional, and societal actors at the global, national and local level have a critical role to play in addressing these structural determinants by redistributing resources and rebalancing power.^12,13^ In particular, local-level actors, with their close connection to community and strong focus on place-based, cross-sectoral action, have been identified as an important context for health equity action.^4,10^ Accordingly, it is important that local actors including local governments, local health agencies, community organizations, and the local community understand what power dynamics are at play and how to address them to promote health equity.

It has been argued that power is one of the most contested concepts in social and political theory, with a plethora of theories and conceptualizations.^14,15^ Structuration theory, developed by Giddens,^16^ is one theory relevant to the health equity policymaking context. Structuration theory explains the dynamic and interdependent relationship between societal structures (institutions, laws, government policies, and societal norms) and the agency of individuals and communities. Structuration theory proposes that on one hand, structural power generated from societal structures can shape or limit individual and community agency, influencing their decisions and the control they have over their lives. On the other hand, individuals and communities can exercise agentic power to challenge and transform inequitable structures. This theory emphasizes the importance of considering the interplay between structural and agentic power, and to address both domains in health equity policymaking. Empowerment theory is another seminal power theory that highlights the importance of increasing the individual's and community's agency and control over their lives. Empowerment theory suggests that increasing individual and community agency requires addressing power imbalances at multiple levels—intrapersonal, interpersonal, and community—simultaneously.^17^ This theory is consistent with a socioecological approach to health, which understands there are multiple, interconnected levels in which to understand and address health inequities, including at the individual-, community- and structural-level.^18^

A number of models and typologies have been developed to help policymakers and practitioners understand how structural and agentic power operate to exacerbate or redress power imbalances in policymaking. Moon's^19^ typology describes eight different types of power (physical, economic, structural, institutional, moral, discursive, expert, and network) available to actors to advance their agendas and safeguard their interests. Moon argues that these forms of power can interact and reinforce one another, shaping inequities in governance and policymaking. Gaventa's Powercube framework provides another approach to understanding power. Gaventa's framework describes the interrelationship between different forms of power (visible, hidden, and invisible) that play out across various spaces (closed, invited, and claimed) and levels (global, national, and local.^20^ Together, Moon and Gaventa's frameworks can assist researchers and policymakers in identifying where and how various types of power are exercised to influence both structural and agentic power.

Local-level actors have the opportunity to address power inequities within and between individuals, communities, and institutions, in both the design of health equity interventions and throughout policymaking processes. While understanding the abovementioned power theories and frameworks is useful, there is currently no comprehensive synthesis of evidence that can help local actors understand how power has been considered and addressed in local-level health equity interventions, representing an important research-practice evidence gap. This study aims to systematically review the international literature (high-income countries) over the last decade to examine how power is addressed in the design and implementation of local health equity interventions, and the reported impacts of these interventions on power imbalances.

## Material and Methods

### Protocol and Search Strategy

The reporting of this systematic review was guided by the Preferred Reporting Items for Systematic Review and Meta-Analysis (PRISMA) Statement.^21^ The search protocol was registered online with PROSPERO on 23 July 2024 (CRD42024572090). A systematic search strategy was developed and applied across five academic research databases: Medline Complete, CINAHL, Global Health, Scopus, and Web of Science. An initial scoping search was developed with a research librarian. The results of this scoping search informed the final search strategy, which included four key concepts: ‘local’, ‘intervention’, ‘health equity’, and power. Synonyms for each of these concepts were identified based on an in-depth familiarity of the literature by the research team and previous studies.^22,23^ Supplemental Material 1 summarizes the search strategy applied to EBSCOhost Medline Complete database. Boolean search operators were adjusted as required for each database. Additional literature sources were identified by hand searching the reference and citation lists of known literature.

### Eligibility Criteria

We included empirical studies and articles that described the development, implementation and/or evaluation of an intervention with an explicit goal to improve health equity (ie, address health inequities generally or improve health for one or more socially or economically disadvantaged population). We were interested in interventions that were implemented at a local geographic level (eg, neighborhoods), including settings within a local neighborhood (eg, schools or recreation center). Only studies that explicitly discussed “power” (or its synonyms) in terms of the intervention design, development, and implementation processes or outcomes were included. Studies were excluded if the intervention indirectly influenced health equity (eg, interventions that increased health equity knowledge and skills in medical clinicians, academics, or governments representatives). We restricted the search to high-income countries due to significant political and social contextual differences across low-, middle-, and high-income countries. Peer-review articles published in English over the last 10 years were included.

### Study Selection

Articles retrieved from the final search strategy on 23 July 2024 were uploaded into Covidence for title and abstract screening based on the eligibility criteria. Title and abstracts were screened by the lead author (SS) for potential relevance. Those considered relevant after abstract screening progressed to full-text review, where each article was assessed against the inclusion and exclusion criteria, independently by two co-authors (SS and AB). Both authors resolved conflicts in Covidence through discussion.

### Theoretical Frameworks

Two frameworks were identified to guide data extraction and analysis. Firstly, the Health Equity Power Framework (HEPF) was developed by Friel and colleagues^24^ to help understand how different actors use different types of power in different ways and in different contexts to influence power inequities in public policy. The HEPF draws together seminal power models and typologies, including Moon's typology of power and Gaventa's Powercube.^19,20^ We have used the framework to analyse how actors (state, market, and civil) use different types (structural, physical, economic, institutional, discursive, moral, expert, and network) and forms of power (visible, hidden, and invisible), across various spaces (closed, invited, and claimed) to either create power inequities or redress them in the local context. We described “actors” using terminology from the original articles instead of using the state, market, and civil constructs of the HEPF, as these were deemed more readily understood by the reader and a more accurate reflection of the primary evidence source. Civil actors include community members, community networks, and community organizations; market actors include private and commercial entities; and state actors include government, government agencies, and other institutional actors. Secondly, VeneKlasen and Miller's Four Expressions of Power typology informed by the work of Dahl was used to analyse how power constructs in the HEPF manifest in power-over, power-with, power-to, and power-within dynamics among individuals, collectives and institutions.^25,26^ Supplemental Material 2 provides definitions for each of the key constructs that informed our data extraction and analysis.

### Data Extraction

Two authors (SS and AB) independently extracted data between 26 August and 13 September 2024, using a standardized template created in Microsoft Excel. All extracted data was cross-checked with disagreements resolved by a third author. Data extracted included bibliographic information (author, year of publication, article title, journal name, abstract), study design (aims, methods, participants, country) and intervention characteristics (intervention name, intervention type, target population, types of actors involved, health equity goals of the intervention, and theories or frameworks that informed the intervention, if stated). Intervention type was classified as health and well-being education programs (designed to teach knowledge and skills related to physical, mental or spiritual health), advocacy education programs (where community members learn knowledge and skills to advocate for health equity causes), community organizing interventions (where community members connect in an organized way to take action on a common cause or injustice), community funding initiatives (where community is given funds and/or decision-making authority over how public or private funds should be spent), community-based health promotion initiatives (prevention-based programs often multisectoral and multifaceted initiatives), community infrastructure projects (which involve local physical assets such as community gardens or parks), and government policies. When interventions involved elements spanning multiple categories (eg, a community funding initiative that also included advocacy education or community organizing), the primary intervention type was determined through team discussion. For example, Sands and colleagues^27^ described mini-grants used to engage the community in transforming local health and fitness environments. While the intervention incorporated broader community engagement/organising strategies, it was classified as a community funding initiative, as the use of grants was the central mechanism. Where applicable, data related to power was extracted and mapped to the constructs of the chosen theoretical frameworks (see Supplemental Material 2).

### Quality Assessment

The Critical Appraisal Skills Programme (CASP) Quality Appraisal Tool–Qualitative Studies Checklist was applied to each included study independently by two co-authors (SS and AB).^28^ The checklist includes 10 questions (answered as *yes*, *no*, *can’t tell*). The aim of quality appraisal in this systematic review was to assess the reporting quality of this body of evidence (it was not used to exclude studies). Results of the appraisal were reported for each study as percent *yes* and percent *no/unclear* for each of the 10 questions.

### Synthesis of Results

We descriptively summarized the frequency (number or percentage) of articles in the search strategy according to key characteristics including country, type of study, and type of intervention. We used thematic analysis to interpret and synthesize extracted data related to power. Thematic analysis allowed for identification, organization and interpretation of findings across multiple studies to answer the research questions. Firstly, SS familiarized with the data by reading and re-reading the extracted text and original articles, noting initial reflections. SS then used an iterative process of generating codes, revisiting the extracted data and articles, and refining codes. SS and CZ reviewed the codes independently and developed draft themes. The research team came together over two meetings to review and refine the themes. Themes are described narratively, with illustrative examples from relevant studies. Some articles discussed interventions related to Indigenous populations, including Aboriginal and Torres Strait Islander peoples, Māori, Native American Indians, and Canadian First Nations peoples. We acknowledge that culturally appropriate terminology for these populations varies by country. When discussing individual interventions, we will use the terminology that was used in the corresponding study, and when reporting results in aggregate, we will respectfully use the term Indigenous populations.

### Reflexivity

The research team has extensive expertise in policy research to promote public health and health equity. We view health inequities as a critical social justice issue that are modifiable through considered policy leadership that is willing to acknowledge and address power imbalances. In this study, we considered unequal power dynamics when analysing data to explore alternative interpretations. We also acknowledge that the systems of oppression our research seeks to understand and disrupt have afforded us degrees of privilege based on our own intersectional positions. To address this status, we adopt a reflexive approach (both in this study and in our broader research practice) to examine our role in sustaining and challenging oppressive systems. We also work to foster similar critical awareness across research and practice and ensure that our policy work remains accessible and actionable for stakeholders with the potential to drive meaningful change.

## Results

We identified a total of 2623 articles through database searches and other sources. Following removal of duplicates and title and abstract screening, 77 articles were eligible for full-text review, of which 39 were excluded. Reasons for exclusion included lack of substantive analysis or discussion of power, ineligible study design (eg, quantitative or review study), ineligible intervention type (eg, medical treatment intervention), and full-text article not available. This resulted in 38 studies for inclusion. See Supplemental Material 3 for the PRISMA flow diagram of included studies.

### Study Characteristics

Table 1 outlines characteristics of the 38 included articles. The majority were conducted in the United States (50%), followed by the United Kingdome (16%), Australia (13%), Canada (11%), New Zealand (8%), and Sweden (3%). Empirical studies that related to a local health equity intervention made up 68% of studies (*n* = 26), while the remaining 32% of articles were descriptions of an intervention without data collection or analysis. A range of intervention types were described including community health education programs (*n* = 8), community organizing interventions (*n* = 8), community funding-based initiatives (*n* = 7), community health interventions (*n* = 4), advocacy education programs (*n* = 4), community-based health promotion initiatives (*n* = 4), community infrastructure projects (*n* = 2), and government policy (*n* = 1). Almost all interventions were targeted, commonly focusing on socioeconomically disadvantaged areas or populations that have been historically and/or systemically excluded, including Indigenous peoples and racial or ethnic minority communities. Assessment of the 26 empirical studies using the CASP Quality Appraisal Tool – Qualitative Studies Checklist found that 77% of studies were rated as being high value to the literature. Reporting rigor was insufficient or unclear for criteria related to the adequate consideration of the relationship between researcher and participants (19% of studies), adequate consideration of ethical issues (19% of studies), and sufficient rigor in data analysis (11% of studies). See Supplemental Material 4 for full results of the quality appraisal checklist.

**Table 1.** Study characteristics (n = 38).

	Study details		Intervention characteristics		
	Author	Country	Name of intervention	Intervention type	Target health equity population
1	Ahmad et al (2017)	Canada	Cancer Awareness: Ready for Education and Screening (CARES) project	Community health intervention	Recent immigrant and low-income communities
2	Alegría et al (2022)	US	LEAP: a civic engagement and leadership program	Advocacy education program	Racially diverse youth in low-income communities
3	Bittle et al (2022)	US	Communities of Focus - participatory budgeting program	Community funding-based initiative	Socioeconomically disadvantaged and racially marginalized communities
4	Butterfield et al (2021)	US	Community Gardens	Community infrastructure project	Not specified
5	Cahuas et al (2015)	Canada	Neighborhood Action (NA) supported by Community Developers	Community organizing initiative	Socioeconomically disadvantaged areas
6	Dodgen et al (2020)	US	SHE Tribe (She's Healthy and Empowered)	Health and well-being education program	Intersectional: Women, low socioeconomic position, racially marginalized
7	Douglas et al (2016)	US	Power U Center for Social Change (Power U) CAAAV Organizing Asian Communities (CAAAV)	Community organizing initiative	African American new and expectant mothers and Asian immigrant and refugee communities
8	Egan et al (2021)	UK	The Big Local initiative	Community funding-based initiative	Socioeconomically disadvantaged areas
9	Firestone et al (2019)	Canada	Niiwin Wendaanimak Four Winds Wellness Program	Health promotion program	Indigenous populations, Toronto
10	Gone et al (2020)	US	Urban American Indian Traditional Spirituality Program	Health and well-being education program	Native American Indian communities, Detroit
11	Haapanen et al (2024)	US	The Amos Project in Cincinnati MOSES in Detroit	Community organizing initiative	Intersectional: Racially diverse, low socioeconomic status
12	Hardt et al (2021)	Australia	Healthier Together	Health and well-being education program	Māori and Pacific Islander families living
13	Haynes et al (2019)	Australia	‘On Track Watch’ (OTW)	Community health intervention	Remote Aboriginal community
14	Heinert et al (2019)	US	CHAMPIONS NETWork	Advocacy education program	Intersectional: Low socioeconomic status, racial minority youth
15	Hikaka et al (2021)	New Zealand	Pharmacist-facilitated medicines review	Health and well-being education program	Maori older adults
16	Ickes et al (2020)	US	Youth Tobacco Advocacy Training (YTAT) program	Advocacy education program	Rural community with weak tobacco control policies/laws
17	Iton et al (2022)	US	Parks4All	Community organizing initiative	Low socioeconomic status areas, racial minority
18	Kerrigan et al (2021)	Australia	Prevention intervention or rheumatic heart disease (RHD) and its precursor, acute rheumatic fever (ARF)	Health promotion program	Aboriginal communities
19	Lansing et al (2023)	US	Innovations 2	Community organizing initiative	Racially diverse, socioeconomically disadvantaged community, Los Angeles
20	Lewis et al (2019)	UK	The Big Local initiative	Community funding-based initiative	Socioeconomically disadvantaged areas
21	Newman Carroll et al (2021)	US	Keep Tobacco Sacred campaign	Health and well-being education program	Native American Indian or Alaska Native student in an Indigenous-serving minority university
22	Perry et al (2016)	UK	National Health Service Health Check program	Community health intervention	Socioeconomically disadvantaged areas
23	Ponsford et al (2021)	UK	The Big Local initiative	Community funding-based initiative	Socioeconomically disadvantaged areas
24	Powell et al (2021)	UK	The Big Local initiative	Community funding-based initiative	Socioeconomically disadvantaged areas
25	Raerino et al (2021)	New Zealand	Te Ara Mua	Community infrastructure project	Maori / Pacific Islander communities (mena whenua)
26	Rämgård et al (2022)	Sweden	Lindängen programme	Community organizing initiative	High immigrant, socioeconomically disadvantaged area
27	Rechis et al (2024)	US	Be Well Communities	Health promotion program	Low-income, medically underserved residents in an area with a high proportion of African American and Hispanic populations
28	Reilly et al (2018)	Australia	Strong Fathers, Strong Families	Health and well-being education program	Aboriginal and Torres Strait Islander fathers
29	Reno et al (2021)	US	Best Babies Zone (BBZ) Initiative	Community organizing initiative	African Americans and Native American Indian populations
30	Sands et al (2014)	US	Call for Partnerships (CfP)	Community funding-based initiative	Socioeconomically disadvantaged areas
31	Schinazi et al (2022)	Canada	CoVivre Program	Community health intervention	Ethnically marginalized populations
32	Shattuck et al (2022)	US	Implementing Strategies to Reduce LGBTQ + Adolescent Suicide	Health promotion program	Intersectional: LGBTQ + intersecting low socioeconomic, and racial minorities areas
33	Simpson et al (2022)	New Zealand	Tuakana–teina Peer-education program for older Māori	Health and well-being education program	Maori older adults
34	Sims et al (2023)	US	Building Healthy Communities Initiative	Community organizing initiative	Unclear
35	Stearne et al (2022)	Australia	Alcohol policy in Mbantua/Alice Springs	Government policy	Aboriginal community
36	Townsend et al (2020)	UK	The Big Local initiative	Community funding-based initiative	Socioeconomically disadvantaged areas
37	Windsor et al (2014)	US	Community Wise	Health and well-being education program	Low-income and predominantly African-American communities
38	Woods-Jaeger et al (2022)	US	Youth Empowered Advocating for Health (YEAH)	Advocacy education program	African American youth and their parents

### How Is Power Considered and Addressed in Local-Level Health Interventions?

Five themes emerged from the literature representing how local-level health equity interventions considered power in their design and implementation (including what aspects of power were silent), and the impact of these interventions on power imbalances. Each theme is described below with an overview of themes and their corresponding codes and example studies detailed in Supplemental Material 5.

1. Increasing knowledge, connectedness, and leadership enhanced individual and community agentic power.
2. Leveraging multiple types of power challenged rigid and inequitable structures and institutional processes.
3. Interventions guided by power-centered frameworks and principles helped rebalance power dynamics.
4. Entrenched structural and institutional power can hinder efforts to rebalance power dynamics.
5. Underrepresentation of government-led interventions in the literature.

*Theme 1: Increasing Knowledge, Connectedness, and Leadership Enhanced Individual and Community Agentic Power*. The literature commonly highlighted interventions that built and fostered individual and community agentic power. These interventions primarily aimed to shift two types of power dynamics: building ‘power-within’ individuals and fostering ‘power-to’ dynamics in community.

Interventions that built ‘power-within’ often used ‘discursive power’ to bolster individual self-worth and self-efficacy, and fostering a sense of belonging and hope. For example, a key strategy used in community organizing interventions involved shifting the public discourse from the dominant biomedical frame of individual responsibility to one that focused on the social determinants of health and structural inequities.^29–31^ These interventions worked to introduce historical context and diverse perspectives regarding the unjust disempowerment of communities that face systematic exclusion to the public discourse (eg, through media, community events, and public meetings) in order to raise institutional and community actors’ awareness of the root cause of health inequities and to challenge ‘invisible power’ related to unjust, dominant ideologies. Other interventions introduced specific strategies to raise critical consciousness among individuals who face unjust health and social inequities by building critical thinking.^32–35^ Providing an enabling environment where community actors critically question, analyse, and reflect on the historical and sociopolitical context that underlies their experience was seen as essential for challenging deeply held beliefs, confronting undeserved feelings of self-blame and shame, and enhancing problem-solving skills within community. Two interventions were designed to build Canadian First Nations peoples’ ‘power-within’ by protecting and lifting ‘spiritual power’ via strategies that enhanced First Nations’ cultural identity and ceremonial knowledge, as well as their coping skills and knowledge of available social supports.^35,36^

Interventions designed to shift ‘power-to’ community used ‘network’ and/or ‘discursive power’ to develop advocacy skills and confidence required for collective impact. Community organizing interventions helped to address isolation, a reported barrier to local health equity action, by creating opportunities for people to connect and share their lived experiences and aspirations, galvanizing collective action. Some community organizing and advocacy education interventions developed community member's leadership, communications, and campaign skills and knowledge of how to create and sustain community collectives, which enabled communities to ‘claim space’ in institutional decision-making processes that were previously inaccessible to them.^29,31,^^37–39^ The United Kingdom's Big Local is an example of a ‘power-to’ initiative, centered on resident-led decision-making, long-term community development, and shifting power to local people. Governance at the local level rests with resident-led partnerships, which have worked to create ‘visible’ spaces for community involvement in decision-making with deliberate efforts to broaden and deepen participation (eg, forming subcommittees open to residents with specific interests and establishing structures such as youth forums to support the involvement of young people).^38^ However, a lack of transparency in rules and procedures within these resident partnerships represented a form of ‘hidden power’ dynamics that impacted participation, essentially excluding some residents from participation.^40^

*Theme 2: Leveraging Multiple Types of Power Challenged Rigid and Inequitable Structures and Institutional Processes*. Shifts in structures and institutional processes that rebalanced power were often the result of actors activating multiple, mutually reinforcing types of powers across different forms and spaces. For example, three community organizing interventions exercised ‘network’, ‘moral’, ‘discursive’, and ‘institutional’ power simultaneously to make ‘visible’ the interests of stakeholders involved in institutional decisions and enable collectives to ‘claim space’ in decision-making processes that occurred in spaces previously ‘closed’ to them. This often involved combining a range of strategies including community engagement, building community networks, message reframing, media campaigns, petitions, and community representation at town hall meetings and public hearings.^31,37^ The impact of these interventions was often significant in terms of shifting power and achieving equity outcomes. The Amos Project in Cincinnati, Ohio, and MOSES (Metropolitan Organizing Strategy Enabling Strength) in Detroit, Michigan, saw faith leaders use ‘moral power’ and ethical framing to reshape narratives (‘discursive power’) and mobilize their congregations to successfully campaign to private and public institutions for action to benefit public education and water equity, respectively.^30^ Another community organizing collective of Chinatown residents in New York ‘claimed space’ by attending ‘visible’ decision-making spaces (eg, town hall meetings) and presenting survey data and community demands to decision-makers, resulting in them securing ongoing tenant association voting rights related to local planning decisions.^30^ Residents in Fresno, California, presented a morally driven public discourse to force a reluctant local government to update their policy to assure investment and maintenance of parks and green spaces in a disadvantaged neighborhood. This same collective then used ‘institutional power’ to fight (and win) a legal motion (strongly contested by the local government) so that a sales tax could be introduced to fund ongoing maintenance of the parks.^37^

We also observed evidence of ‘expert’ and ‘economic power’ in funding initiatives. For example, a local government and local health department implemented participatory budgeting, where community members were brought into ‘visible’, formal decision-making spaces, invited to review data, determine priorities and develop strategies for addressing health inequities, and were given full control over how the public funds would be spent.^41^ This participatory budgeting initiative, along with other funding-based programs, recognized community members as experts in understanding their own needs, granting them a degree of ‘expert power’ and ‘economic power’, both of which marked a significant departure from typical institutional practices.^27,41,42^ Some interventions reported that such shifts in power dynamics required a level of trust and partnership with community that was new, unfamiliar, and often uncomfortable for institutional actors. This discomfort reflected a form of ‘invisible power’, rooted in the internalized norm that institutions are the primary holders of expertise. Implementing strategies that build community capacity in planning, budgeting, leadership and governance were reported as ways institutional actors attempted to address this discomfort and support shifts in expert and economic power.^27,42^

*Theme 3: Interventions Guided by Power-Centered Frameworks and Principles Helped Rebalance Power Dynamics*. Interventions that considered power in their design, implementation, or evaluation were often underpinned by frameworks that explicitly acknowledged power imbalances and provided strategies to address them. These frameworks offered an opportunity to identify, acknowledge, and challenge both ‘invisible power’ (embedded in societal and institutional norms) and ‘hidden power’ within institutional processes, in order to prioritize and elevate community voices. Supplemental Material 6 summarizes the key power-related theories and frameworks that guided interventions in this review.

Empowerment theory was used in several interventions, directing strategies to act at the individual, organization, and institutional levels simultaneously.^27,31,43^ For example, a message reframing strategy involved the dissemination of information to community residents, community organizations, and policymakers to describe the harmful impact of inequitable rezoning and housing policies on Chinatown resident health and well-being.^31^

Decolonizing theory was used to acknowledge and address ‘structural and systemic power’ imbalances associated with colonization in intervention design and intervention development processes.^33,44^ For example, the Strong Fathers, Strong Families program explicitly recognized colonization and dispossession as significant historical forces that disrupted the traditional roles of Aboriginal and Torres Strait Islander men within families and communities, limiting their capacity to engage meaningfully as father figures, providers, leaders, and decision-makers and leading to sustained disempowerment.^44^ As a result, the education program sought to address key challenges such as shame and lack of confidence among fathers in accessing and participating in antenatal and early childhood services, in conjunction with standard parenting education.

Critical consciousness theory aims to enhance policymaker and community actors’ critical thinking and critical reflection skills to recognize and challenge learned ways of thinking and blind acceptance of oppressive systems (‘invisible power’).^32,34^ For example, the Youth Empowered Advocating for Health (YEAH) intervention taught youth to critically examine and recognize how structural racism shapes their lives and communities, then built on this awareness to equip them with advocacy and policy change skills to address these issues.

Other strengths-based frameworks, such as Kaupapa Māori theory and self-determination, center Indigenous knowledge, privilege Indigenous voices, and promote Indigenous autonomy in intervention design and implementation.^35,^^44–46^ For example, the Kaupapa Maori framework enabled meaningful engagement with Indigenous groups in an urban indigenization project, with Māori participants reporting they felt equal in decision-making: “We are not in a queue, we are a partner, not a stakeholder”.^45^ However, the ‘hidden power’ of institutional actors to control whose voices are included and how frameworks are implemented can undermine their impact. For example, an Australian study highlighted that despite national and global recognition of the importance of self-determination in policies that affect Indigenous peoples, self-determination was absent in policy processes for the development of an alcohol policy, which reinforced power inequities experienced by First Nations Australians in a local community.^47^

Codesign approaches, such as community-based participatory research (CBPR), were adopted in several interventions.^33,48^ Similar to other power-centered frameworks, codesign offers a framework to equitably engage community members as experts, acknowledging and amplifying the value of their diverse contributions and increasing their power.^49^ Applying bidirectional learning principles (also known as co-learning or two-way learning) was identified as an effective strategy to equalize partner voices and elevate community as experts in intervention design.^33,50^ For example, bidirectional learning was used to facilitate knowledge and insights to flow equally between academic staff and students regarding Native American traditional use of tobacco and public health approaches to tobacco prevention. This resulted in a policy change to the tobacco prevention policy that achieved public health goals, while also respecting tobacco's place in Native American culture.^50^

*Theme 4: Entrenched Structural and Institutional Power Can Hinder Efforts to Rebalance Power Dynamics*. Entrenched ‘structural power’ and ‘institutional power’ either actively or passively moderated community's power in several health equity interventions. At a structural level, socioeconomic disadvantage and structural racism diluted community's ‘network power’ by affecting the capacity of community actors to participate in interventions—shaping both the breadth (number and diversity of individuals involved) and depth (extent and nature of how community can get involved) of participation.^37,38^ Another study showed how systems of homophobia manifested in how parents and the broader community had ‘power-over’ school administration and teachers, who feared negative professional repercussions if they supported the implementation of a school-based initiative to promote health and well-being for LGBTQI students.^51^ Another study demonstrated the potential of unintentional harmful impacts of ‘structural power’ inequities on community actors’ psychological health. For example, an intervention that increased community actors’ awareness and knowledge about structural racism and oppression resulted in individuals experiencing feelings of hopelessness and powerlessness.^39^

Explicit attempts by institutional actors to silence community ‘network power’ via policy, legal, and economic mechanisms were also reported in studies. For example, local public officials used their ‘institutional power’ to attempt to prevent a community organizing intervention from running an advertising campaign on public buses that drew attention to inequitable public investment in green spaces between lower and higher socioeconomic areas.^37^ When the same community organizing group secured enough votes to force the city to implement a sales tax for ongoing park maintenance, the public office attempted to change the interpretation of voting rules so the motion would not pass. Residents were then required to initiate and fund a lawsuit, which ultimately ruled in their favor.

In a less explicit example, institutional actors also exercised ‘hidden power’, controlling policy processes from ‘backstage’ by creating barriers to meaningful participation or keeping issues on or off the agenda. For example, community members were employed by an institution to help connect institutional decision-makers with community to inform local-level policy decisions, however, the community liaisons reported feeling like they were “walking a delicate tight rope” or that they were “one decision away from being fired” if they supported resident priorities and interests that diverged from the interests of the more powerful institutional actors.^52^

*Theme 5: Underrepresentation of Government-Led Interventions in the Literature*. Despite holding significant ‘structural’ and ‘institutional power’, local governments were notably underrepresented in the literature reviewed. Only three interventions were led by local authorities, revealing a striking absence of explicit engagement with power dynamics in local health policymaking.^41,47,52^ Interventions were diverse, including a participatory budgeting initiative that handed over decision-making authority to community members,^53^ an alcohol policy in a regional Australian community,^47^ and an intervention that employed members of the community to connect those in positions of power (eg, government officials) with community members usually excluded from decision-making.^52^

## Discussion

In this systematic review of 38 studies, we found that power can be rebalanced in multiple ways through the design and implementation of local-level health equity interventions. Interventions that were reported as increasing individual and community agentic power used strategies to increase community actors’ knowledge and interconnectedness to build ‘power-within’ individuals, as well as developing leadership, critical thinking, and advocacy skills to build ‘power-to’ community. While there was less evidence of ‘structural power’ being rebalanced compared to agentic power, interventions that were able to make equity-positive shifts in structural power occurred when multiple types of powers, including ‘network’, ‘discursive’’, moral’, ‘economic’, and ‘expert’ power, were activated simultaneously by different actors. Interventions that utilized power-centered frameworks in their design and implementation helped to equalize power dynamics by helping actors acknowledge and address structural and agentic power imbalances. ‘Structural power’ inequities and the deliberate misuse of ‘institutional power’ limited rebalancing of power dynamics. Importantly, we found silences in the literature, including a lack of local government-led interventions.

Our finding that local health equity interventions commonly target individual-level agentic power aligns with broader public health research showing that, despite widespread recognition of structural determinants in health inequities, health and social interventions predominantly focused on interpersonal and intrapersonal change.^54,55^ This may be partly attributed to the fact that most included studies came from neo-liberal welfare states, which can favor targeted, individual-level programs and interventions rather than structural policies to address health inequities. Nevertheless, consistent with structuration theory, we found interventions that build and enhance community actors’ agentic power can lead to equity-positive changes to structural power dynamics. For example, our review found that interventions using ‘discursive power’ to reframe health inequities from issues of individual responsibility to those related to social determinants and structural barriers, enabled both community actors and policymakers to shift their focus toward actions that addressed the structural causes of inequities. The importance of challenging and changing dominant frames shaped by inequitable systems and structures is highlighted by Bacchi's ‘What's the Problem Represented to Be?’ (WPR) framework.^56^ Bacchi argues that policies and programs are not neutral responses to problems but are shaped by how problems are represented or constructed. When health equity problems are not framed as structural problems, a form of ‘invisible power’ is activated, which can result in marginalized community actors internalizing dominant ideologies, blaming themselves for the inequities they experience and preventing them from challenging the status quo. Our review also identified interventions that strengthened community actors’ leadership skills and capacity to build and sustain collectives (eg, community organizing initiatives). These interventions activated ‘network power’, enabling communities to ‘claim space’ in institutional decision-making processes that had previously excluded them, and rebalance structural power inequities.

We identified that interventions, such as community organizing, achieved equity-positive shifts in ‘structural power’ when actors combined mutually reinforcing forms of power across multiple levels and spaces. Community organizing is a promising but underused strategy for advancing health equity. A review of community organizing in public health found that organizing can shift public health priorities toward community needs, build local leadership and power, and support policy change on social determinants of health.^57^ Aligned with empowerment theory and public health approaches, these cases illustrate how diverse actors, both community-based and institutional, can mobilize power across interconnected levels to disrupt and dismantle inequitable structures.^17,18^ Importantly, these examples emphasize a distinction in the health equity literature between ‘giving power’ and ‘gaining power’.^58^ ‘Giving power’ occurs when structurally stronger actors grant power to others, but the extent to which structurally weaker actors become empowered depends on their capacity to gain and retain that power. For instance, if a local government forms a steering group for decision-making but retains final authority, they maintain power over the steering group, limiting both the scope and nature of the power granted. This review showed how community organizing initiatives resulted in structurally weaker actors (eg, community members) gaining power in decision-making spaces that had previously been ‘closed’ to them.^31,37^ Evidence shows that designing interventions that enable communities to gain power requires a higher level of trust and partnership skills that institutional actors are often not typically used to.^59^ Trust is an ongoing process that requires actors to make themselves vulnerable to and/or dependent on others, and this shift in power dynamic can create discomfort.^60^ Researchers have emphasized that actors involved in health equity interventions should view discomfort not as a barrier to trust and partnerships, but as a normal, constructive, and essential aspect of achieving transformational social change.^29,61^

The importance of underpinning health equity interventions with frameworks that explicitly acknowledge and address power imbalances was highlighted in multiple studies in our review. Codesign principles grounded in power-centered theories and frameworks (eg, empowerment theory, decolonizing theory, critical consciousness theory, radical healing framework) equipped actors with the understanding and tools to reflect on the root cause of inequities, such as privilege, power, and historic injustices, identify inequitable power dynamics, and provide guidance on how to prioritize the voices, knowledge, and epistemologies of people with less ‘structural power’. Principles of self-determination guided interventions developed with and for Indigenous populations. The United Nations Declaration on the Rights of Indigenous Peoples acknowledge that Indigenous peoples’ right to self-determination, including the right to have genuine decision-making authority over their lives, governing systems, and economic, social, and cultural development is enshrined in international law.^62^ Interventions grounded in self-determination imply an inherent shift of power from dominant colonial structures to Indigenous peoples. However, studies identified in our review found that self-determination principles were not always applied in practice. For example, the development of alcohol policy in Central Australia found an absence of First Nations Australians in policy development, reinforcing the need for increased effort by governments to implement the right to self-determination in policymaking. We also found that adopting principles of bidirectional learning in the development and implementation of interventions can also help actors equalize power dynamics. Literature from the field of global health recommend bidirectionality as a means to support mutual capacity building and to shift, mitigate, and/or question systems and power hierarchies in community engagement and policymaking.^63,64^ While several models have been developed to support bidirectional learning, scholars highlight several barriers to its effectiveness in addressing power hierarchies, including structural racism and discrimination, inadequate time and funding commitment to implementing bidirectional principles, and the failure to acknowledge diverse knowledge systems, epistemologies, and perspectives.^64,65^

We identified studies that outlined how ‘structural power’ inequities, such as structural racism, homophobia, and socioeconomic disadvantage, inhibited the capacity of actors to activate ‘network power’ and ‘expert power’.^37,38,51^ Studies also described how institutions deliberately misused ‘institutional power’ to block community actions related to a health equity intervention, which triggered community actors to activate ‘moral power’ and publicly highlight the injustice.^36^ Previous research suggests that increasing political will for equitable policymaking will require political leadership and advocacy movements to use ‘moral power’ as a means to argue for fairness and social justice.^63^

The burden of addressing ‘structural power’ imbalances should not rest with those who experience health inequities. Local governments and other local institutional actors have the opportunity to use their policies, services, laws, and public spending levers to generate structural and systemic change that elevates the power of community members disproportionately burdened by adverse health outcomes. However, our review found that few interventions were situated within government contexts. This may partly reflect an underrepresentation of government-led interventions in academic literature, compared to community-based initiatives that involve researchers. Nonetheless, there is an urgent need for policymakers to turn their gaze inward and take explicit action to rebalance power within their policies and policymaking processes. Government actions often do not shift power to communities experiencing health inequities, in part due to their structural alignment with market and corporate interests. Governments frequently prioritize economic goals over health equity, creating significant barriers to redistributive change. This highlights the importance of community organizing as a means of building civil society power to counterbalance economic influence and advocate for more equitable policies.

As noted earlier in the discussion, the included studies predominantly described time-bound, targeted interventions rather than more permanent structural-level policies, both of which are essential to reduce health inequities. One approach governments can adopt is proportionate universalism, whereby policies are universal in scope but implemented with a scale and intensity proportionate to the level of need.^66^ This approach aims to reduce the social gradient in health by ensuring benefits for all, while directing additional support (potentially through local-level interventions) to those experiencing greater disadvantage.

### Revised Health Equity Power Framework

**Figure 1.**
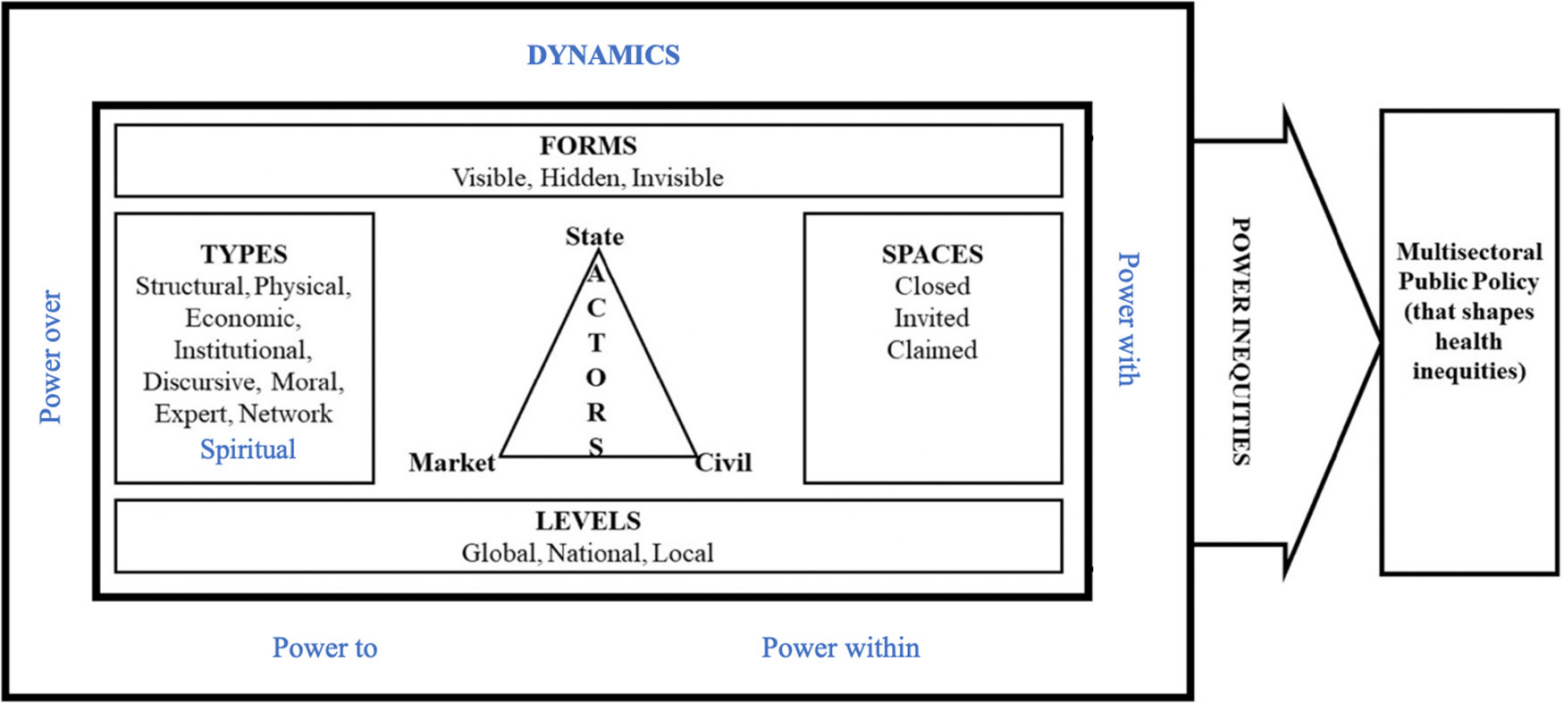
Revised health equity power framework (Revised from 24, 25).

In this review, we utilized the HEPF alongside the Four Expressions of Power typology to analyse power in local-level health equity interventions.^24,25^ Integrating the Four Expressions typology into the HEPF provided a novel and valuable lens for understanding how the different types, forms, spaces, actors, and levels of power (as described in the HEPF) manifested as power-over, power-with, power-to, and power-within dynamics to influence power inequities in policymaking. In addition, we found that spiritual power was explicitly referenced in First Nations Canadian interventions and implicitly for other Indigenous population interventions. As spiritual power is not currently a construct in the HEPF, our findings suggest that its addition could be important, particularly when examining power dynamics in health equity interventions involving Indigenous populations. Therefore, we propose an adaption to the current HEPF to incorporate the four expressions of power dynamics and the addition of ‘spiritual power’ as a construct that influences power inequities in policymaking. A revised framework is presented in Figure 1.

### Strengths and Limitations

This systematic review was theory-informed and guided by a transparent and rigorous systematic review process to examine a broad range of literature related to power in local-level health interventions across high-income countries. Our comprehensive focus on power theory makes a novel contribution to research methods that can advance our understanding of how we practically analyse and address power imbalances. Notwithstanding, the inclusion criterion may have missed studies where power was not explicitly described or where alternative terminology, such as working at the ‘local level’, fell outside the scope of our search strategy. Working toward establishing consistent terminology for analysing power in health equity interventions would benefit future reviews. Almost a third of the studies were descriptive accounts of interventions, and even among those that included evaluations, few explicitly measured the effect of interventions on power dynamics. Future research on local health equity interventions could incorporate explicit measures of power to better understand how interventions influence power imbalances and contribute to health equity outcomes.

## Conclusion

This systematic review is the first to examine how to shift power in local-level health equity interventions implemented in high-income countries. Our analysis provides practical guidance and examples for how policymakers, other institutional actors, and community actors can consider and rebalance power dynamics to strengthen equity in their policies and programs. However, the responsibility must not rest solely on communities that experience systematic discrimination and health inequities. Local governments, supported by higher levels of government, and other institutions with local power must take explicit and deliberate action to rebalance power in policies and policymaking processes.

## Supplemental Material

sj-docx-1-joh-10.1177_27551938251401131 - Supplemental material for Addressing Power in Local-Level Policies and Programs to Reduce Health Inequities – A Systematic Review Supplemental material, sj-docx-1-joh-10.1177_27551938251401131 for Addressing Power in Local-Level Policies and Programs to Reduce Health Inequities – A Systematic Review by Sally Schultz, Anne Barrow, Sharon Friel, Christina Zorbas, Anna Peeters and Kathryn Backholer in International Journal of Social Determinants of Health and Health Services
