# Supplementary material for: Addressing Power in Local-Level Policies and Programs to Reduce Health Inequities – A Systematic Review
Source: Int J Soc Determinants Health Health Serv. 2025 Dec 1;56(2):189–202. doi: 10.1177/27551938251401131 (PMC12987999; doi:10.1177/27551938251401131)
Supplement: sj-docx-1-joh-10.1177_27551938251401131 - Supplemental material for Addressing Power in Local-Level Policies and Programs to Reduce Health Inequities – A Systematic Review [file sj-docx-1-joh-10.1177_27551938251401131.docx]

Supplemental Material 1: Search terms applied to EBSCOHost Medline

| Concept | Search terms |
| --- | --- |
| Local | Local OR Municipal* OR Shire OR Provence OR Place OR Neighbourhood OR Neighborhood OR Community |
| Intervention | Intervention OR Polic* OR Program* OR Strateg* OR Plan OR Framework |
| Health Equity | “Health equit*” OR “health inequit*” OR “health disparit*” OR “health inequal*” OR “social determinant*” |
| Power | Power OR Empower* OR Autonomy OR Voice OR Agen* OR Transformat* OR Redistribut* OR Inclusi* OR Rebalanc* OR “Self determination” OR “Procedural fairness” OR “Distributive justice” OR “Relational justice” |

**Supplemental Material 2: Definitions of power constructs that informed data extraction and analysis** (Friel et al., 2021; Giddens, 1984; VeneKlasen & Miller, 2002)

| **Structure and Agency**  *Structural power*: power generated from institutions, laws, government policies, and societal norms that can shape and/or limit agency and decisions of individuals and collectives.  *Agentic power:* the influence of actors (individual or collectives) over or with other actors.  **Health Equity Power Framework**  Types:  *Structural:* Structures of society which shape the actions or thinking of actors, and influence the functioning of institutions.  *Institutional:* The creation or use of rules and decision-making procedures to shape thinking and action in order to achieve a specific purpose.  *Economic:* The use of material resources (e.g., money) to shape the thinking and actions of other actors, and influence the functioning of institutions.  *Physical:* The use, or threat of use, of physical force by an actor to shape the thinking or actions of other actors.  *Discursive:* Discursive/ideational power is wielded when actors shape the language others use to conceptualize, frame, and thereby define and understand an issue.  *Moral:* Moral power is wielded when actors shape the principles that others believe to be right or wrong, and the actions that may then follow.  *Expert:* The legitimizing of knowledge, and influencing what people understand to be factually true or correct.  *Network:* Network power is wielded when actors use their personal relationships with others to shape their thinking and/or action.  Forms:  *Visible:* Interests which are visible in public spaces or formal decision-making arena e.g. parliaments, government bodies, consultative forums, and civil society gatherings.  *Hidden:* By controlling the policy processes from ‘backstage’, powerful actors create barriers to participation, resulting in some actors being excluded from the decision-making table or certain issues kept off the agenda.  *Invisible:* Through the internalisation of dominant ideologies, norms and values, people unconsciously.  Spaces:  *Closed:* Arenas where decisions are made behind closed doors often by an elite group of actors without broader consultation or involvement.  *Invited:* Invited spaces are also controlled by elite actors but they do invite others in to discuss issues of mutual interest. The elite actors control the meeting agenda and minutes, and can use these to reflect their interests.  *Claimed:* Spaces that less structurally powerful actors create through lobbying to influence policy and gain a seat at the table, and in formal and informal gatherings outside of the institutionalized policy arenas.  Actors:  *Civil*: community members, networks and organizations  *Market:* private, commercial entities  *State:* government, government agencies  Levels: Power is found across different levels of decision-making and authority including *local*, *national* and *global*. This review was focused at the local level.  **Four Expressions of Power**  *Power-over*: describes the dominant approach in public policymaking, characterized by force, coercion, domination and control.  *Power-with*: refers to a power dynamic based on respect, mutual support, solidarity and collaborative decision-making, typically aligned with co-design and participatory approaches.  *Power-to*: denotes the generative potential of power - the power to make a difference, create something new, or achieve goals and joint action. For example, via the development of citizen leadership and advocacy skills.  *Power-within*: pertains to a person's sense of self-worth and self-efficacy, enabling individuals to believe in their ability to harness their 'power to' and 'power with' to effect change. |
| --- |

Supplemental Material 3: Preferred reporting items for systematic reviews and meta‐analyses (PRISMA) flow diagram of included studies


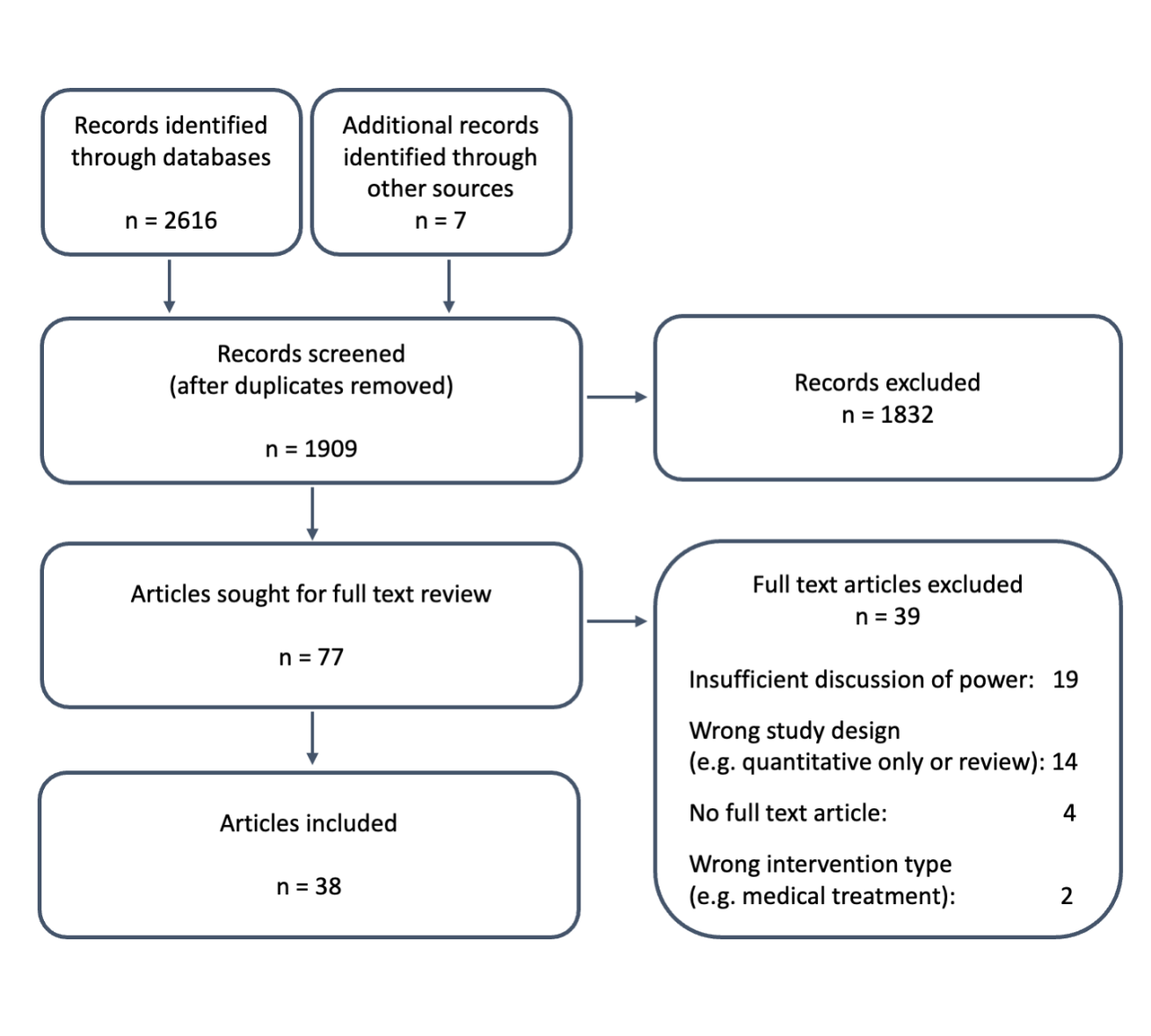


**Supplemental Material 4**: **Reporting of Critical Appraisal Skills Programme (CASP) Quality Appraisal Tool - Qualitative Studies Checklist**

*Note: Only empirical studies were assessed. Articles that described an intervention were reported as N/A*

| **Author, year** | **Clear statement of aims of research** | **Appropriate qualitative methodology** | **Appropriate research design** | **Appropriate recruitment strategy** | **Appropriate data collection** | **Adequate consideration of relationship between researcher and participants** | **Adequate consideration of ethical issues** | **Sufficient rigor in data analysis** | **Clear statement of findings** | **Value of research** |
| --- | --- | --- | --- | --- | --- | --- | --- | --- | --- | --- |
| Ahmad et al. (2017 | Yes | Yes | Yes | Yes | Yes | Yes | Yes | Yes | Yes | High |
| Alegría et al. (2022) | Yes | Yes | Yes | Yes | Yes | Yes | Yes | Yes | Yes | Yes |
| Bittle et al. (2022) | N/A | N/A | N/A | N/A | N/A | N/A | N/A | N/A | N/A | N/A |
| Butterfield et al. (2021) | Yes | Yes | Yes | Yes | Yes | Yes | Can't tell | Yes | Yes | Moderate |
| Cahuas et al. (2015) | Yes | Yes | Yes | Yes | Yes | Yes | Can't tell | Can't tell | Yes | High |
| Dodgen et al. (2020) | N/A | N/A | N/A | N/A | N/A | N/A | N/A | N/A | N/A | N/A |
| Douglas et al. (2016) | N/A | N/A | N/A | N/A | N/A | N/A | N/A | N/A | N/A | N/A |
| Egan et al. (2021) | Yes | Yes | Yes | Yes | Yes | Yes | Yes | Yes | Yes | High |
| Firestone et al. (2019) | Yes | Yes | Yes | Yes | Yes | Yes | Yes | Yes | Yes | High |
| Gone et al. (2020) | N/A | N/A | N/A | N/A | N/A | N/A | N/A | N/A | N/A | N/A |
| Haapanen et al. (2024) | Yes | Yes | Yes | Yes | Yes | Yes | Can't tell | Yes | Yes | High |
| Hardt et al. (2021) | Yes | Yes | Yes | Yes | Yes | Yes | Yes | Yes | Yes | High |
| Haynes et al. (2019) | Yes | Yes | Yes | Yes | Yes | Yes | Yes | Yes | Yes | High |
| Heinert et al. (2019) | N/A | N/A | N/A | N/A | N/A | N/A | N/A | N/A | N/A | N/A |
| Hikaka et al. (2021) | Yes | Yes | Yes | Yes | Yes | Yes | Yes | Yes | Yes | Moderate |
| Ickes et al. (2020) | Yes | Yes | Yes | Yes | Yes | Yes | Yes | Yes | Yes | High |
| Iton et al. (2022) | N/A | N/A | N/A | N/A | N/A | N/A | N/A | N/A | N/A | N/A |
| Kerrigan et al. (2021) | Yes | Yes | Yes | Yes | Yes | Yes | Yes | Yes | Yes | High |
| Lansing et al. (2023) | Yes | Yes | Yes | Yes | Yes | Yes | Yes | Yes | Yes | High |
| Lewis et al. (2019) | Yes | Yes | Yes | Yes | Yes | Can't tell | Yes | Yes | Yes | Moderate |
| Newman Carroll et al. (2021) | N/A | N/A | N/A | N/A | N/A | N/A | N/A | N/A | N/A | N/A |
| Perry et al. (2016) | Yes | Yes | Yes | Yes | Yes | Can't tell | Yes | Yes | Yes | Moderate |
| Ponsford et al. (2021) | Yes | Yes | Yes | Yes | Yes | Can't tell | Yes | Yes | Yes | High |
| Powell et al. (2021) | Yes | Yes | Yes | Yes | Yes | Can't tell | Yes | Yes | Yes | High |
| Raerino et al. (2021) | Yes | Yes | Yes | Yes | Yes | Can't tell | Yes | Yes | Yes | High |
| Rämgård et al. (2022) | Yes | Yes | Yes | Yes | Yes | Yes | Yes | Can't tell | Yes | High |
| Rechis et al. (2024) | N/A | N/A | N/A | N/A | N/A | N/A | N/A | N/A | N/A | N/A |
| Reilly et al. (2018) | Yes | Yes | Yes | Yes | Yes | Yes | Yes | Yes | Yes | High |
| Reno et al. (2021) | Yes | Yes | Yes | Yes | Yes | Can't tell | Can't tell | Yes | Yes | Yes |
| Sands et al. (2014) | N/A | N/A | N/A | N/A | N/A | N/A | N/A | N/A | N/A | N/A |
| Schinazi et al. (2022) | N/A | N/A | N/A | N/A | N/A | N/A | N/A | N/A | N/A | N/A |
| Shattuck et al. (2022) | Yes | Yes | Yes | Yes | Yes | Can't tell | Can't tell | Yes | Yes | High |
| Simpson et al. (2022) | Yes | Yes | Yes | Yes | Yes | Yes | Yes | Yes | Yes | High |
| Sims et al. (2023) | Yes | Yes | Yes | Yes | Can't tell | Can't tell | Yes | Can't tell | Yes | Moderate |
| Stearne et al. (2022) | Yes | Yes | Yes | Yes | Yes | Yes | Yes | Yes | Yes | High |
| Townsend et al. (2020) | Yes | Yes | Yes | Yes | Yes | Yes | Yes | Yes | Yes | High |
| Windsor et al. (2014) | Yes | Yes | Yes | Yes | Yes | Yes | Yes | Yes | Yes | High |
| Woods-Jaeger et al. (2022) | N/A | N/A | N/A | N/A | N/A | N/A | N/A | N/A | N/A | N/A |

**Supplemental Material 5: Themes, codes and corresponding studies**

| **Theme** | **Codes** | **Example of a corresponding study** |
| --- | --- | --- |
| 1. Increasing knowledge, connectedness and leadership enhanced individual and community agentic power | - Interventions aimed to build hope, self-worth, and a sense of belonging, (power within) - Interventions shifted the narrative (discursive power) from dominant individual responsibility to enhancing community understandings of structural power (invisible power), like structural racism, recognising how it manifests in their lives and communities - Interventions developed skills, confidence and opportunities to help community claim or be invited into spaces (power-to) - Positive feedback loops were observed when interventions increased collective capability, which led to further action/opportunities, which further increased capabilities, and so on. | Windsor et al., 2014  Haynes et al., 2019  Woods-Jaeger et al., 2024  Firestone et al., 2019  Alegría et al., 2022  Gone et al., 2020  Sims et al., 2023  Egan et al., 2021 |
| 2. Leveraging multiple types of power challenged rigid and inequitable structures and institutional processes | - Community organising activated network, discursive and moral power to advocate for and achieve changes to policies and institutional processes - Funding initiatives resulted in shifts in expert and economic power to community | Douglas et al., 2016  Iton et al., 2022 |
| 3. Interventions guided by power-centred frameworks and principles helped rebalance power dynamics | - Helps institutional stakeholder embrace community expert power and avoid tokenistic engagement - Creates genuine power-with dynamics - Helps both policymakers and community recognise and challenge learned ways of thinking and blind acceptance of oppressive systems - Has potential to reduce the potential of disempowerment and harm to those who face marginalisation, however governments need to do more, especially regarding implementing self-determination in policymaking. - Helps to embrace Indigenous epistemologies and elevate Indigenous voices - The use of these frameworks was most prominent in community education, community health programs and community organising interventions - Frameworks included Empowerment theory, Indigenous frameworks (e.g. Self-determination, Decolonising theory, Māori frameworks), Critical consciousness, and Co-design frameworks supported by bi-directional learning principles | Haynes et al., 2019  Reilly and Rees, 2018, Douglas et al., 2016  Woods-Jaeger et al., 2024  Windsor et al., 2014  Firestone et al., 2019 Raerino et al., 2021  Simpson et al., 2022  Hikaka et al., 2021 |
| 4. Entrenched structural and institutional power can hinder efforts to rebalance power dynamics | - Structural determinants (including SEP), oppressive systems such as racism and homophobia) and governance structures were specifically called out as moderating power via its impact on participation (breadth and depth) and inclusion, as well as having unintentional negative impacts on individual health and wellbeing (e.g. feelings of hopelessness / powerlessness) - There were explicit attempts to silence community network power (visible power) - Community ‘brokers’ employed by public officials to bridge the gap between community and institution were limited by fear of ‘rocking the boat’ (job security and future funding) (invisible power) | Lewis et al., 2019  Iton et al., 2022  Shattuck et al., 2022  Alegría et al., 2022  Cahuas et al., 2015 |
| 5. Silences and opportunities to address power in health equity interventions | - Only three studies were within the local government context - One intervention described the use of moral power to expose the social injustice related to the health equity issue | Stearne et al., 2022  Bittle, 2022  Cahuas et al., 2015  Iton et al., 2022 |

**Supplemental Material 6: Definitions of power-centred frameworks identified in the review**

| *Empowerment theory*: Promotes consideration of power dynamics at the individual, organization, and community level simultaneously to increase individual and community control and promote social transformation.  *Critical consciousness theory*: Involves a reflective awareness of power and privilege in order to understand social inequities and take critical action towards social change.  *Decolonizing theory:* Acknowledges the historical and detrimental effects of colonization on Indigenous communities, privileging and honouring Indigenous voices, knowledges, and epistemologies.  *Self-determination*: Recognizes and respects Indigenous knowledge systems, requires Indigenous people to have power and control over decisions that affect themselves and their communities, be able to practice culture and have freedom and dignity in their lives.  *Kaupapa Māori:* emphasises Indigenous epistemologies and knowledges, and promotes transformation through self-determination.  *Radical Healing Framework:* Encourages the acknowledgement of and active resistance from oppression, while also visioning paths towards healing.  *Community-based participatory research (CBPR):* Equitably involves community members, researchers, and other actors in the research process, recognizing the unique strengths that each bring.  *Bidirectional learning principles:* Commitment to a reciprocal process of cocreating knowledge (both-way learning) to equalise power dynamics in codesigned interventions. |
| --- |
